# Breaking Barriers: Prospective Study of a Cohort of Advanced Chronic Obstructive Pulmonary Disease Patients To Describe Their Survival and End-of-Life Palliative Care Requirements

**DOI:** 10.1089/jpm.2018.0363

**Published:** 2019-02-22

**Authors:** Daniel Gainza-Miranda, Eva Maria Sanz-Peces, Alberto Alonso-Babarro, María Varela-Cerdeira, Concepción Prados-Sánchez, Guadalupe Vega-Aleman, Ricardo Rodriguez-Barrientos, Elena Polentinos-Castro

**Affiliations:** ^1^ Palliative Homecare Team Northern Area of Madrid, SERMAS, San Sebastian de los Reyes, Spain.; ^2^ Palliative Care Department, La Paz Hospital, Madrid, Spain.; ^3^ Pneumology Department, La Paz Hospital, Madrid, Spain.; ^4^ Preventive Medicine, Public Health, SERMAS, San Sebastian de los Reyes, Spain.; ^5^ Investigation Support Unit for Primary Care, Gerencia Atención Primaria, Madrid, Spain.; ^6^ Investigation Support Multidisciplinary Unit for Primary Care and Community North Area of Madrid, Madrid, Spain.

**Keywords:** ACP, end of life, palliative care, place of death, pulmonary disease chronic obstructive, quality of life

## Abstract

***Background and Aim:*** Consensus has been reached on the need to integrate palliative care in the follow-up examinations of chronic obstructive pulmonary disease (COPD) patients. We analyzed the survival from the initiation of follow-up by a palliative home care team (PHCT) and described the needs and end-of-life process.

***Setting and Design:*** This study was a prospective observational cohort study of advanced COPD patients referred to a PHCT. Sociodemographic variables, survival from the start date of follow-up using the Kaplan–Meier model, health resource consumption, perceived quality of life, main symptomatology, opioid use, and advanced care planning (ACP) were analyzed.

***Results:*** Sixty patients were included. The median survival was 8.3 months. Forty-two patients died at the end of the study (85% at home or in palliative care units). The most frequent cause of death was respiratory failure in 39 patients (93%), with 29 of these patients requiring sedation (69%). Dyspnea at rest, with an average of 5 (standard deviation [SD] 2) points, was the main symptom. Fifty-five patients (91%) required opioids for symptom control. The median score in the St. George's Respiratory Questionnaire was 72 (SD 13). The mean number of visits by the home team was 7 (SD 6.5). The mean number of admissions during the monitoring period was 1.5 (SD 0.15).

***Conclusions:*** The characteristics of the cohort appear suitable for a PHCT. The follow-up care provided by our multidisciplinary unit decreased the number of hospitalizations, favored the development of ACP, and enabled death at home or in palliative care units.

## Introduction

Chronic obstructive pulmonary disease (COPD) is currently the fourth leading cause of death in Western countries.^1^

The advanced stage of the disease is characterized by a high frequency of symptoms, loss of functionality, and a great number of exacerbations, leading to a significant deterioration in the patient's quality of life. This deterioration is similar or even higher than the final stage of advanced cancer patients.^2–4^ However, access to specific palliative care resources for COPD patients remains low.^5,6^ The literature indicates that multiple barriers are observed in the access of these patients to palliative care.^7^ A proper description of the final phase of this disease may allow for a better assessment of patients' needs and their care and treatment.^8^

The difficulty in establishing the prognosis and recognition of the final stage of life may represent one of the main causes of nonreferral to palliative care services.^7,9^ Therefore, the main consensus guidelines for the management of COPD patients include the collection of prognostic criteria to allow for the identification of end-of-life patients.^10–12^ Other authors recommend starting an early palliative care approach for the disease regardless of its prognosis.^13^

Patients with advanced COPD are generally in a home environment and require hospital care during flare-ups. Pneumology, palliative care, and primary care departments should work in a coordinated manner to ensure the end-of-life continuity of care. However, there barely exist works on the most effective health structure for attending to end of life for these patients, nor on the natural background of patients with advanced COPD.^14,15^ These kinds of studies can provide us with information about symptomatic burden, quality of life, and real needs of health resources for these patients. This information would let us develop more effective health structures and improve the care that these patients receive, offering them and their families more realistic expectations as the disease progresses and they approach the moment of death.

A multidisciplinary unit for the care of patients with advanced respiratory problems that integrated the pneumology department and palliative care unit was created in our hospital in 2013. Residential and hospital services are included in the palliative care unit, which are responsible for contact with the primary care doctors. The HOLD study^16^ sought to describe the trajectory at the end of life in actual clinical practice in patients seen by a palliative home care team (PHCT) integrated into a multidisciplinary unit, and the results allow for measures to be taken to improve the care model.

We analyzed the survival from the start of monitoring by a PHCT that had been in place for four years and described the end-of-life needs and processes of these patients. Other specific goals of this work were as follows: to describe the main symptoms and quality of life, to estimate the use of opioids in standard clinical practice for treating dyspnea, and to evaluate the use of health resources in advanced stages of COPD patients monitored at home.

## Materials and Methods

### Design

This study was a prospective observational cohort study of advanced COPD patients referred by the pneumology department, palliative care support team, or primary care team for monitoring by the PHCT.

The inclusion criteria were as follows: older than 18 years, COPD Global Initiative for Chronic Obstructive Lung Disease (GOLD) grade IV,^11^ and functional deterioration with a palliative performance scale (PPS) <60. The exclusion criteria were as follows: cognitive impairment or severe mental illness, a diagnosis of lung cancer or cystic fibrosis, inability to receive home care because of the absence of a primary caregiver and inability to understand Spanish.

Patients diagnosed with lung cancer during the monitoring period or those who moved out of the study area were also excluded. All patients provided written consent for their participation in the study.

Once a patient was included in our program, monthly monitoring visits up to a two-year maximum were carried out by the PHCT in addition to the usual clinical care. In case of urgent hospital admission, the attendance of the patients in the program was carried out by a support team or the pneumology service, both included in the multidisciplinary unit, to guarantee continuity of care. During the follow-up, the palliative care unit could request a hospice admission if deemed necessary. Other PHCT key components are shown in Table 1. The monitoring protocol and the scope of the study were extensively described in a previous work by our group (HOLD study).^16^

**Table 1.** Palliative Home Care Team Key Components in the Multidisciplinary Unit

1. Attendance at monthly meetings of the multidisciplinary unit to agree on treatments and care plans for new patients and update treatment goals for patients in the program.
2. Monthly scheduled domiciliary visits by PHCT and telephone support and nonscheduled domiciliary visits as needed.
3. Primary care support with joint domiciliary visits and telephone consultations.
4. Disease treatment optimization, including education and management of inhaler therapy, domiciliary oxygen therapy, and written exacerbation plans.
5. Holistic and systematic assessment of symptoms with special attention to dyspnea with comprehensive management of refractory breathlessness, including nonpharmacological strategies (such as breathing techniques, recovery breathing positions, and the use of a handheld fan) and written instructions for the use of opioids prescribed.
6. Early access to hospice services to avoid hospital or emergency department admissions.
7. Routine discussion regarding goals of care and advanced care planning.

PHCT, palliative home care team.

The ethics committee approved the study with the project code Pi-2011.

### Variables

The survival time of the patient cohort from the inclusion in the study to the date of death was calculated as the main variable. The date and place of death, reason for death, need for palliative sedation (defined as the use of specific sedatives to relieve intolerable suffering from refractory symptoms by reducing a patient's level of consciousness),^17^ clinical reason for sedation, and the implementation of advanced care planning (ACP) were recorded for the end-of-life description.

The following secondary variables were studied.

#### Sociodemographic variables

Data on age, gender, and education level were recorded at the start of monitoring.

#### Variables related to clinical characteristics

The following data were recorded: forced expiratory volume in one second (FEV1) in last spirometry, use of chronic domiciliary oxygen therapy and noninvasive mechanical ventilation (NIMV), number of admittances in the year before the start of monitoring, BODE Index,^18^ Charlson Comorbidity Index,^19^ PPS,^20^ Barthel Index (BI),^21^ body mass index, and level of physical activity using the short version of the International Physical Activity Questionnaire.^22^

#### Quality of life

St. George's Respiratory Questionnaire (SGRQ) was self-administered and supervised on the first visit to the patient's home and quarterly during the follow-up.^23^

#### Symptom load and care

The Edmonton Symptom Assessment System (ESAS)^24^ and the dyspnea grade according to the Modified Scale of the Medical Research Council (mMRC) were recorded at every home visit.^25^ The percentage of patients with an opioid prescription during monitoring to control dyspnea and the average doses were also recorded.

The number of visits by the PHCT and phone calls made to the team by patients or their families during the monitoring period was determined via clinical records. We also collected the numbers of visits to the emergency department (ED) and hospital admissions during the year before the start of monitoring and during the monitoring.

### Statistical analysis

Most of the objectives were described via descriptive statistics that summarized the categorical variables using absolute and relative frequencies. Continuous variables with a normal distribution were analyzed using the mean and standard deviation (SD), and continuous variables with an asymmetrical distribution were analyzed using the medians and interquartile ranges (IQR). The survival curves were generated using the Kaplan–Meier model. The survival assessment was reported with a confidence interval of 95% (95% CI). Dependent *t*-test was used to evaluate the difference in admittances and visits to the ED before and after the start of the follow-up by PHCT.

The statistical program used was Stata (StataCorp.2013. Stata Statistical Software: Release 13; StataCorp LP, College Station, TX).

## Results

A total of 66 advanced COPD patients were referred to the PHCT between January 1, 2014, and February 1, 2017. Six patients were excluded because they did not meet the inclusion criteria (five due to PPS >60 and one due to of GOLD grade III). Five of the 60 included patients (8%) were discharged during monitoring because of functional improvement, and one patient refused to continue in the study.

Most of the patients in our study were males (48). The mean age was 73 years (SD 12). The FEV1 was 26.8 (SD 4.3). Table 2 shows the remaining demographic and clinical characteristics at the start of monitoring.

**Table 2.** Demographic and Clinical Variables at the Start of Monitoring

*Variable (*n* = 60)*	*Total*
Age: mean (SD)	73.8 (12.2)
Gender: male (%)	80
Level of studies (%)	
Without studies	6.6
Vocational training	20
Primary	48.3
Secondary	11.6
University	13.3
FEV1 (SD)	26.8 (4.3)
BODE score (%)	6 (3.8)
	7 (27)
	8 (11.5)
	9 (23.1)
	10 (34.6)
Physical activity (%)	
Medium	5
Low/sedentary	95
NIMV (%)	31.6
No. of admittances in the year before the start of monitoring/after monitoring: mean (SD)	2.5 (1.57)/1.5 (0.15), *p* < 0.01
Number of visits to the emergency department in the year before the start of monitoring/after monitoring: mean (SD)	3.5 (2.01)/0.8 (1.04), *p* < 0.01
PPS: mean (SD)	51.66 (9.7)
Barthel Index: mean (SD)	69.41 (24.8)
Charlson Index: mean (SD)	2.5 (1.65)
Home oxygen therapy prescription (%)	100
BMI (SD)	22 (4.4)
BMI <21 (%)	<21 (42)

BMI, body mass index; FEV1, forced expiratory volume in one second; NIMV, noninvasive mechanical ventilation; PPS, palliative performance scale; SD, standard deviation.

**FIG. 1.**
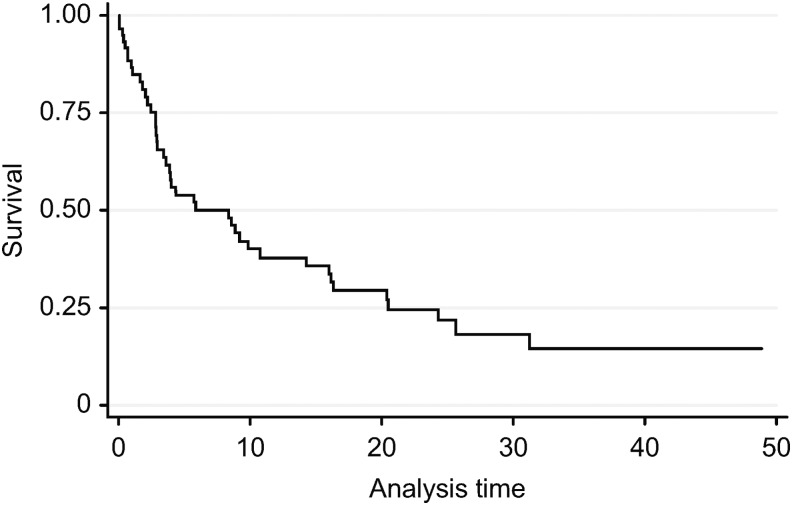
Kaplan–Meier survival curve.

**FIG. 2.**
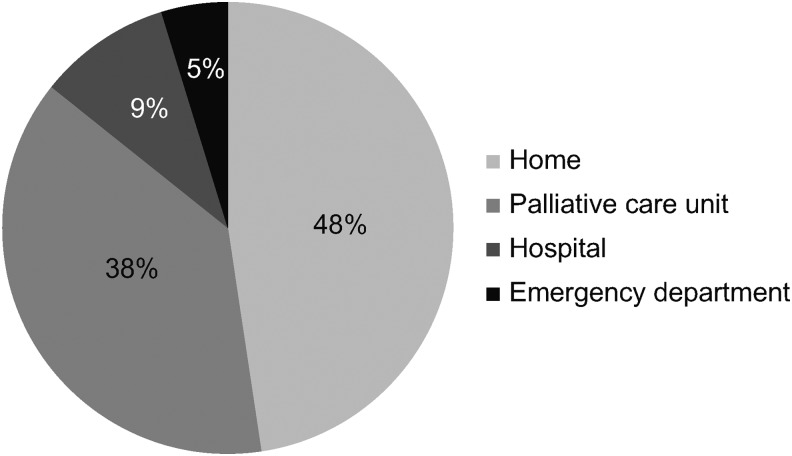
Place of death.

The median survival of patients from the start of monitoring by the unit was 8.3 months (95% CI 2.7–20.5) (Fig. 1). A total of 42 (70%) patients died. Thirty-six of these patients (85%) died at home or in palliative care units (Fig. 2). The cause of death was respiratory failure in 39 patients (93%) and complications related to comorbidity in the remaining 3 patients (7%). Twenty-nine patients (69%) required palliative sedation, including in the hospital and home area. Dyspnea was the main cause for palliative sedation in 24 patients (83%). Other reasons were agitation in three patients (10%) and existential distress in two patients (7%).

At the beginning of the follow-up, none of the 42 deceased patients had performed ACP. During the follow-up, 23 patients (55%) had performed ACP. Nine patients (21%) did not perform ACP due to the short time of follow-up (less than three visits), while eight patients (19%) did not wish to discuss ACP and two patients (5%) did not perform ACP due to conspiracy of silence.

**FIG. 3.**
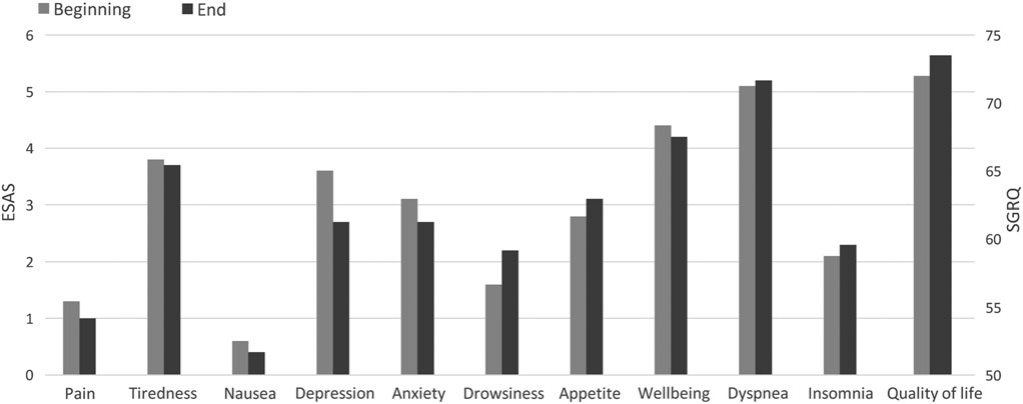
Evolution of symptoms and quality of life. ESAS, Edmonton Symptom Assessment System; SGRQ, St. George's Respiratory Questionnaire.

Fifty-four patients (90%) had grade IV dyspnea according to the mMRC, and the remaining six (10%) patients had dyspnea grade III. Dyspnea at rest was the symptom with the highest score in the ESAS. Main results of ESAS and SGRQ during the follow-up are summarized in Figure 3.

Fifty-five (91%) patients required opioids to control their dyspnea, and morphine was the opioid used in 54 cases (90%). The mean equivalent daily dose of morphine (EDDM) was 14.88 mg (SD 14) of basal opioids, and the mean rescue EDDM was 6.21 mg (SD 12.5). At the dying phase, the mean EDDM was 44.8 mg (SD 16.5).

The median number of visits by the PHCT was 4.5 (IQR 2–20). Forty-nine patients (82%) made calls to the PHCT, and the median number of calls from these patients was 4 (IQR 2–6).

The mean number of admittances and emergency visits in the year before the monitoring and after monitoring is shown in Table 2.

## Discussion

The HOLD study is one of the first cohort studies of patients with advanced COPD who were followed by a PHCT. Our results show that our chosen inclusion criteria are feasible for joint monitoring by palliative care teams according to the high symptom load and a limited life expectancy showed by our cohort.

The profiles of the patients in the unit are consistent with the profile of patients who were considered in end-of-life stage by the European Respiratory Society^9^ and the descriptions of end-of-life profiles in different publications.^12^ The survival rate of the cohort was 8.3 months. These patients were subsidiary for follow-up by specific palliative care teams according to the literature.^7,9,26,27^ Different published reviews indicated that early palliative care should be started in parallel to active treatment and monitoring by specific teams must be requested when uncontrolled symptoms are observed and for ACP, which must be preferably performed in the final stages of the disease.^28,29^

In a study similar to ours conducted in Canada, Horton et al.^30^ published results on the intervention of a palliative care team in a cohort of patients with moderate or severe COPD. The profile of the 30 recruited patients revealed an important degree of obstruction with a dyspnea grade that was somewhat lower than that of our cohort (resting dyspnea in 60% of patients), and home oxygen therapy was observed in half of their patients. The study did not offer a survival median, although half of the patients died after one year of monitoring and exhibited a symptom load and deterioration of quality of life similar to that in our study.

In another study conducted in Australia, Smallwood et al.^15^ published results of a cohort of 171 patients with advanced COPD followed up by the integrated respiratory and palliative care team. Patients also revealed an important degree of obstruction and minimal exertion dyspnea in 75% of patients. Sixty-two patients of the cohort died with a survival median of 12.1 months (7.8–27.1). No symptom control or quality-of-life measures were reported.

The main cause of death in our patients was respiratory insufficiency, which was followed to a much lesser degree by secondary complications to the comorbidity. However, in many studies, patients with COPD generally die because of comorbidity, especially morbidity caused by cardiovascular causes and cancer.^31,33^ This result may be explained because the comorbidity of our cohort was not very high, which was likely because most of the patients were included from the pneumology outpatient clinics, where the patient profile may be different from that in other areas. However, in studies focused on populations with advanced COPD, the main cause of death is respiratory failure, which was also the case in our study.^34,35^ Dyspnea was the main symptom listed at the first visit, and the diagnosis was primarily minimal effort or rest dyspnea. Tiredness, loss of well-being, and depression were other prevalent symptoms in our cohort. These findings are consistent with most studies published on advanced COPD patients.^36,37^ Most patients required the use of opioids to control dyspnea during monitoring. The doses used were adjusted to the recommendations in terms of safety for the control of dyspnea in patients with advanced COPD^38^ and were similar to that of previous studies.^39,40^ In contrast, several studies show the underuse of opioids among pneumologists.^41,42^

High scores in the SGRQ were obtained at the start and similar results were found after three months of follow-up despite the progression of disease. Our results in terms of loss of quality of life were far superior to that of other cohort studies of patients with COPD.^43^ This difference was likely related to performance in patients at earlier stages of the disease.

The main places of death were home and palliative care unit (PCU), which covered 85% of deaths. These results are consistent with the results of Smallwood et al., in which only 24.6% of patients died in an acute hospital bed. Boland et al.^44^ also found that 63% of patients died at home or in the PCU, which was somewhat lower than that of our study.

A recent study that analyzed the place of death in 14 countries^45^ found that the percentage of death at home ranged from 54% in Mexico to 10.4% in Canada. Spain exhibited an intermediate percentage (36%). The differences are explained by the presence of a primary caregiver, the monitoring by palliative care teams, the development of ACP, age, and cultural factors that favor death at home. These data together with a different organization of patient monitoring may explain our higher percentage of deaths in the home and PCU.

None of our patients had performed ACP before referral. However, ACP was undertaken in half of the patients during the follow-up. These data are inconsistent with several studies,^15,46^ where communication with end-of-life patients with COPD was reported as poor. In contrast, Sinclair et al.^47^ systematically performed end-of-life conversations and obtained percentages similar to our study. The undertaking of ACP is facilitated by participation in the monitoring of palliative care teams as previously described.^48^ Earlier referral to the palliative care team could improve percentage of ACP in the future as nine patients (21%) with no ACP had a less than three-month follow-up. We also found that eight patients (19%) did not wish to discuss ACP. Similar percentages were also found in the literature.^49^

A total of 69% of patients required palliative sedation. To ensure appropriate use of palliative sedation, the PHCT followed a previously created checklist.^17^ Our group and others have described a much lower percentage of patients requiring palliative sedation at home.^17^ However, data are not available for sedation at the end of life in patients with COPD. A systematic review of studies on sedation at home that included almost exclusively oncological patients obtained percentages between 1% and 72%.^50^ A high frequency of symptoms of poor control justifies the higher percentages of sedation.^51^ Caraceni et al.^52^ performed a retrospective study in a tertiary Italian hospital and obtained a rate of 68% sedation, which was similar to our own results. The presence of dyspnea as the most prevalent symptom and the main reason of sedation in our study may help explain our high percentage of sedation.

The literature describes that the number of hospitalizations and ED visits increases with the approach of end of life of COPD patients.^53^ This increase was not observed in our study, and the number actually decreased from the previous year. This result may be related with the involvement of specific palliative care teams in patient monitoring, as it was reported by at least two systematic reviews.^54,55^

Our study has several limitations. First, most of the patients in the study were included from pneumology consultations, which could affect certain results. Nevertheless, the inclusion of patients from other areas would not change the main conclusions of our study. Second, this was also a small, long-term, single-group, cohort study and so there was no separate control group, which may limit the generalization of the results. There are no previous studies that describe the end of life in the home setting with which we can compare our results. A “before and after” comparison was undertaken to investigate any associations between PHCT care and unscheduled health care use. Future cluster multicenter randomized studies should be done to rigorously test the effectiveness of the PCHT follow-up.

Therefore, we conclude that the patient profile of our PHCT had a survival of eight months, exhibited a high symptom load, and presented a low quality of life during the follow-up and low levels of physical and functional activity, consistent with the profile of patients who require the attention of a specific palliative care team. Monitoring by our PHCT appears to favor the development of ACP and a smaller number of hospitalizations to enable death at home or in palliative care units.
